# Diabetic Retinopathy Treated with Laser Photocoagulation and the Indirect Effect on Glycaemic Control

**DOI:** 10.1155/2014/158251

**Published:** 2014-07-17

**Authors:** Anna Praidou, Sofia Androudi, Periklis Brazitikos, George Karakiulakis, Eleni Papakonstantinou, Ioannis Tsinopoulos, Stavros Dimitrakos

**Affiliations:** ^1^St. Paul's Eye Unit, Royal Liverpool University Hospital, L7 8XP, UK; ^2^Department of Ophthalmology, Aristotle University, 54124 Thessaloniki, Greece; ^3^Department of Pharmacology, School of Medicine, Aristotle University, 54124 Thessaloniki, Greece

## Abstract

*Purpose*. To identify any possible relation between glycaemic control and previous laser photocoagulation for diabetic retinopathy. *Methods*. Seventy-two patients with diabetes were included in the study and were separated into 2 groups according to previous treatment (group A) or not (group B) with argon laser photocoagulation. Glycaemic control was estimated by measuring blood levels of HbA1c in four consecutive measurements. *Results*. Blood levels of HbA1c in group A were significantly lower 3, 6, and 12 months after laser treatment as compared to blood levels of HbA1c before laser treatment (7.1 ± 0.4% versus 7.6 ± 0.9%, 7.2 ± 0.2% versus 7.6 ± 0.9%, and 7.1 ± 0.2% versus 7.6 ± 0.9%, resp., all *P* < 0.05). Blood levels of HbA1c in group B did not differ significantly in four consecutive measurements. *Conclusion*. Our results suggest that we should anticipate a better glycaemic control in cases of patients with diabetes previously treated with laser photocoagulation.

## 1. Introduction

Diabetic retinopathy (DR) is the most common microvascular complication of diabetes, which can result even in blindness [1]. DR can be classified into two categories: nonproliferative (NPDR) and proliferative (PDR) [2]. PDR occurs with severe retinal ischemia and is characterized by the growth of new blood vessels on the optic disc or elsewhere in the retina [2]. Diabetic macular edema (DME) can occur at any stage of DR and is regarded as the principal cause of vision loss in patients with diabetes [2]. Retinal hypoxia is implicated in the pathogenesis of DME and in the development of retinal neovascularization. Hypoxia results in increased expression of vascular endothelial growth factor (VEGF), which is the most potent inducer of increased vascular permeability and the trigger for the formation of abnormal and leaking new vessels [3]. In previously published studies we have already shown the well-established involvement of vascular endothelial growth factor (VEGF) and the contribution of other growth factors [4] in the pathogenesis of PDR [5] and NPDR with DME [3].

Clinical trials have also shown the effectiveness of laser photocoagulation, vitrectomy, and control of hyperglycemia and hypertension for DR [1]. In this study we examined the hypothesis that previous treatment with argon laser photocoagulation in patients with diabetes is positively related to their glycaemic control.

## 2. Patients and Methods

The study was conducted prospectively in 2009-2010 at the Aristotle University of Thessaloniki, Greece, following the tenets of the Declaration of Helsinki. Approval of the Institutional Review Board Ethics Committee of the Medical School of Aristotle University of Thessaloniki was also obtained. All patients signed an informed consent after the purpose of the study was explained in detail to each subject.

Patients with diabetes were included in the study and were separated into 2 groups according to treatment (group A) or not (group B) with argon laser photocoagulation. Inclusion criteria for both groups included (i) patients with type 2 diabetes mellitus, (ii) visual acuity ranging between 20/40 and 20/70 for each eye of the same subject, (iii) NPDR with DME diagnosed with biomicroscopy and confirmed on fundus fluorescein angiography (FFA). Exclusion criteria for both groups included (i) previous treatment with laser photocoagulation, (ii) no evidence of PDR or clinically significant macular edema (CSME) on biomicroscopy or FFA.

All patients with diabetes included in the study were offered laser treatment after receiving counseling by the same retinal specialist related to their management of DR and were provided information leaflet regarding the laser treatment and were treated in the same hospital by the same retinal specialist. Glycaemic control was estimated by measuring blood levels of HbA1c (Hemoglobin A1c) in four consecutive measurements, that is, at baseline and at 3 months, 6 months, and 12 months after laser treatment in group A and in four consecutive measurements at baseline and at 3, 6, and 12 months in group B as well.

All values were expressed as the mean ± standard error (mean ± SE). Statistical analysis was performed with the Statistical Package for the Social Sciences (SPSS). The differences between groups were analyzed by multivariate analysis of variance (MANOVA). The differences between groups with respect to sex were tested by chi-square test (Table 1). A two-tailed *P* value of less than 0.05 was considered to indicate statistical significance.

## 3. Results

Seventy-two patients with diabetes were included in the study: group A (treated with laser) 36 patients and group B (untreated) 36 patients. Characteristics of patients included in the study are shown in Table 1. There were no significant differences between the two diabetic groups with respect to sex, age, duration of diabetic disease, and blood levels of HbA1c prior to treatment with argon laser photocoagulation.

Furthermore, there were also no significant differences between the two groups with respect to comorbidities such as systemic arterial hypertension (SAH), dyslipidemia, renal dysfunction and atherosclerotic cardiovascular disease, their income, educational level, habits (smoking and/or drinking), and insurance status (private versus social).

Blood levels of HbA1c in group A were significantly lower 3 months after laser treatment as compared to blood levels of HbA1c before laser treatment (7.1 ± 0.4% versus 7.6 ± 0.9%, *P* < 0.05, MANOVA). Interestingly, blood levels of HbA1c in group A sustained significantly decreased after 6 months after laser treatment as compared to blood levels of HbA1c before laser treatment (7.2 ± 0.2% versus 7.6 ± 0.9%, *P* < 0.05, MANOVA). More interestingly, blood levels of HbA1c in group A sustained significantly decreased even after 12 months after laser treatment as compared to blood levels of HbA1c before laser treatment (7.1 ± 0.2% versus 7.6 ± 0.9%, *P* < 0.05, MANOVA). No significant negative correlations between the extent or the type (focal, grid, or both) of laser photocoagulation and the concentration of HbA1c in blood after laser treatment were found in group A (all *P* values > 0.05). Blood levels of HbA1c in group B did not differ significantly in four consecutive measurements (7.5 ± 0.8% versus 7.6 ± 0.9%, 7.4 ± 0.8% versus 7.6 ± 0.9%, and 7.4 ± 0.6% versus 7.6 ± 0.9%, resp., all *P* > 0.05) (Table 2).

No significant positive correlations were found between blood pressure (BP) levels, body mass index (BMI), weight, and the laser treatment. But there were positive correlations between some social habits, such as smoking, alcohol, exercise, and the laser treatment but not significant ones.

## 4. Discussion

Panretinal (PRP) and focal or grid laser photocoagulation, when indicated in patients with PDR or DME, respectively, has beneficial effect on DR by reducing the risk of severe visual loss even more than 50% [6–8]. In our study, we came to an interesting observation that previous argon laser photocoagulation in patients with diabetes is positively related to their glycaemic control. A feasible explanation for this trend is that patients with diabetes who undergo laser photocoagulation treatment apparently consider laser photocoagulation as an operation and subsequently attain the attitude of the operated patient, who in general complies better to doctor's guidelines, such as antidiabetic treatment, exercise and diet, achieving stricter glycaemic control, and hypothesis that was generally supported from our Institutional Psychologist.

Additionally, the improved glycaemic control could be attributed to a psychological effect of becoming motivated to improve the health status [9] after having been subjected to laser photocoagulation and a stark realization of the diabetic disease that is leading to impaired eyesight. With the inherent fear of vision loss in this patient population some had probably experienced some degree of visual loss and may fear losing more, which could motivate them to change their behaviour. Also the discomfort or pain associated with the procedure [10] could be a potential motivator to alter diet to prevent the likelihood of requiring further courses of treatment.

On the other hand, patients with diabetes who reject the laser treatment obviously do not realize the seriousness of their eye involvement (either as a result of their own perception or because of a patient-doctor communication failure) and thus continue to their previous way of living (in terms of glycaemic control, systemic follow-ups with their diabetologists, healthy eating).

We also suggest that diabetologists have an additional reason to encourage patients with diabetes to visit an ophthalmologist [11], expect for direct treatment of DR [3, 6, 8]; they should expect better glycaemic control in certain cases. We are not aware if the same results for better glycaemic control imply for patients who had laser for PDR; to our experience, because PDR does not always imply severe visual loss, patients with PDR do not always realize the seriousness of DR eye disease.

## 5. Conclusion

To our knowledge, this is the first study that examines the possible “indirect effect” of laser treatment to glycaemic control. Larger number of patients and duration of glycaemic control could safely examine more related parameters and support our findings.

## Figures and Tables

**Table 1:** Baseline characteristics (mean ± standard error) of patients with diabetes included in the study.

	Group A (treated)	Group B (untreated)
Total patients (*n*)	36	36
Age (yrs)	62 ± 2.5	65 ± 1.8
Duration of diabetes (yrs)	14 ± 3	14 ± 1.5
HbA1c (%) at baseline (g/dL)	7.6 ± 0.9	7.6 ± 0.9
Systolic BP (mmHg)	165 ± 9	160 ± 11
Weight (Kg)	82 ± 10	80 ± 12
BMI (Kg/m^2^)	32 ± 6	32 ± 4

**Table 2:** HbA1c (g/dL) blood levels (mean ± SE) in both groups at baseline and at 3 consecutive measurements after laser treatment.

	Group A (treated)	Group B (untreated)
Baseline	7.6 ± 0.9%	7.6 ± 0.9%
Month 3	7.1 ± 0.4%	7.5 ± 0.8%
Month 6	7.2 ± 0.2%	7.4 ± 0.8%
Month 12	7.1 ± 0.2%	7.4 ± 0.6%
